# Long-term treatment-free remission in CML after early dasatinib discontinuation due to interstitial pneumonitis

**DOI:** 10.1007/s12185-026-04241-y

**Published:** 2026-06-30

**Authors:** Makoto Kashimura, Yuki Fujioka, Yong-Mei Guo, Teruko Takahashi, Keiichi Moriya, Naoto Takahashi

**Affiliations:** 1 Division of Hematology, Shinmatsudo Central General Hospital, Matsudo, Japan; 2 Division of Central Laboratory, Akita University Hospital, Akita, Japan; 3 Department of Hematology, Nephrology and Rheumatology, Akita University Graduate School of Medicine, Akita, Japan

**Keywords:** Chronic myeloid leukemia, Dasatinib, Treatment-free remission, Immune surveillance, NK-like T-cell

## Abstract

Achieving treatment-free remission (TFR) in patients with chronic myeloid leukemia (CML) requires several years of tyrosine kinase inhibitor (TKI) therapy and a sustained deep molecular response (DMR). We report an exceptional case of a 54-year-old woman who achieved long-term TFR despite discontinuing dasatinib after only 9 months due to grade 3 interstitial pneumonitis. At the time of discontinuation, the duration of DMR was 128 days. Following cessation, BCR::ABL1 transcript levels transiently rebounded to 0.0729% on the International Scale but subsequently declined spontaneously without resumption of TKI therapy. The patient has remained in TFR for more than 7 years. Immunological analysis revealed clonal expansion of a unique CD56^+^CD57^+^CD8^dim^ T-cell population possessing natural killer (NK)-like properties, as confirmed by T-cell receptor gene rearrangement analysis. This specific clone persisted after discontinuation, suggesting a role in the sustained immune surveillance of residual leukemia stem cells. These findings suggest that the quality of immune reconstitution, specifically the induction of innate-like effector T-cell clones, may be a more critical determinant of TFR success than the absolute duration of therapy.

## Introduction

The achievement of a sustained deep molecular response (DMR) is widely recognized as a prerequisite for attempting treatment-free remission (TFR) in patients with chronic myeloid leukemia (CML) [1, 2]. Current clinical guidelines, such as those provided by the National Comprehensive Cancer Network (NCCN), European LeukemiaNet (ELN), and Japanese Society of Hematology (JSH), generally recommend at least 3 years of tyrosine kinase inhibitor (TKI) therapy and a minimum of 2 years of sustained DMR before discontinuation [3–5]. These criteria were established to ensure that the residual leukemia stem cell (LSC) pool was sufficiently reduced to prevent molecular relapse.

However, accumulating evidence suggests a more complex reality: Some patients successfully achieve TFR even when they do not meet the stringent conventional criteria [6]. Furthermore, the observation that TFR can be maintained in certain individuals despite the persistence of detectable minimal residual disease (MRD) strongly implies the involvement of an endogenous immune-mediated control mechanism [7, 8]. Although various immune effectors, including natural killer (NK) cells and cytotoxic T lymphocytes, have been implicated in immune surveillance [9], the precise immunological landscape that allows safe TKI discontinuation, especially after a very short duration of therapy, remains to be fully elucidated.

Herein, we report on a unique and instructive case of CML. The patient maintained TFR for > 7 years despite discontinuing dasatinib after only 9 months of therapy because of severe drug-induced interstitial pneumonitis. This case, characterized by an exceptionally short DMR duration of only 128 days, provides a rare opportunity to examine the role of a CD56^+^CD57^+^CD8^dim^ T-cell clone in achieving a stable TFR outside the bounds of traditional guidelines.

## Case report

A 54-year-old Japanese woman presented to our hospital with leukocytosis detected during a routine health checkup. There was no significant medical history, and she had never smoked. She was asymptomatic, and physical examination revealed no abnormalities such as splenomegaly or superficial lymphadenopathy. Laboratory data at the initial visit are summarized in Table 1. Cytogenetic analysis of bone marrow confirmed the presence of the Philadelphia chromosome. The Sokal and ELTS scores were classified as low risk, and no additional cytogenetic abnormalities were identified. Based on these findings, the patient was diagnosed with chronic phase CML and started on dasatinib (100 mg/day).

**Table 1:** Laboratory data at the initial presentation

Complete blood count		Biochemistry and Serology		Bone marrow examination	
WBC	29.4 × 10^9^/L	AST	30 U/L	NCC	759,000/μL
RBC	2.94 × 10^12^/L	ALT	23 U/L	MegK	258/μL
Hb	15.2 g/dL	G-GTP	58 U/L	Blast	1.6%
Ht	48.4%	LDH	461 U/L	Promyelo	4.4%
Plt	360 × 10^9^ /L	T.Bil	0.5 mg/dL	Myelo	15.2%
Ret	2.0%	BUN	11.6 mg/dL	Meta	4.4%
Blast	0%	sCre	0.54 mg/dL	Stab	30.6%
Promyelo	0%	UA	9.4 mg/dL	Seg	26.2%
Myelo	1.5%	Na	141 mEq/L	Baso	1.2%
Meta	6.5%	K	4.5 mEq/L	Eos	6.0%
Stab	4.5%	Cl	103 mEq/L	Mon	0.2%
Seg	68.5%	Ca	9.3 mg/dL	Lyn	4.4%
Baso	5.0%	iP	3.3 mg/dL	Plasma	0.4%
Eos	0%	CRP	0.12 mg/dL	EBl	5.0%
Mon	3.5%	CMV IgG	not done	Karyotype	46,XX,t(9;22) [19/20]
Lyn	10.5%	CMV IgM	not done		

WBC, white blood cells; RBC, red blood cells; Hb, hemoglobin; Ht, hematocrit; Plt, platelets; Ret, reticulocytes; AST, aspartate aminotransferase; ALT, alanine aminotransferase; G-GTP, gamma-glutamyl transpeptidase; LDH, lactate dehydrogenase; T Bil, total bilirubin; BUN, blood urea nitrogen; sCre, serum creatinine; UA, uric acid; Na, sodium; K, potassium; Cl, chloride; Ca, calcium; iP, inorganic phosphorus; CRP, C-reactive protein; NCC, nucleated cell count; MegK, megakaryocytes; Promyelo, promyelocytes; Myelo, myelocytes; Meta, metamyelocytes; Stab, band neutrophils; Seg, segmented neutrophils; Baso, basophils; Eos, eosinophils; Mon, monocytes; Lyn, lymphocytes; Plasma, plasma cells; EBL, erythroblasts; CMV, cytomegalovirus; IgG, immunoglobulin G; IgM, immunoglobulin M

**Fig. 1:**
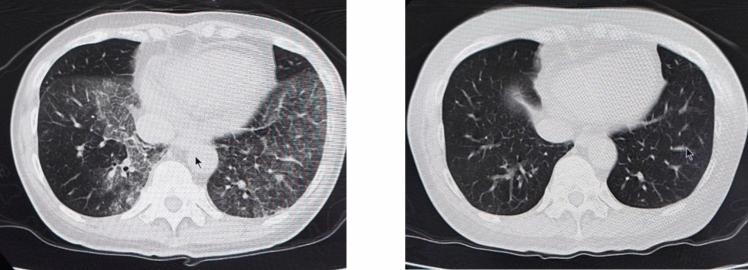
Chest computed tomography (CT) findings of drug-induced interstitial pneumonitis. July 13, 2018 (day 282): CT images obtained at the time of visiting the emergency department for cough and dyspnea. Diffuse bilateral interstitial opacities consistent with dasatinib-induced interstitial pneumonitis were observed (A). Post-steroid Treatment: Following dasatinib discontinuation and prednisolone administration, the interstitial opacities rapidly improved and completely resolved 60 days after discontinuation (B)

**Fig. 2:**
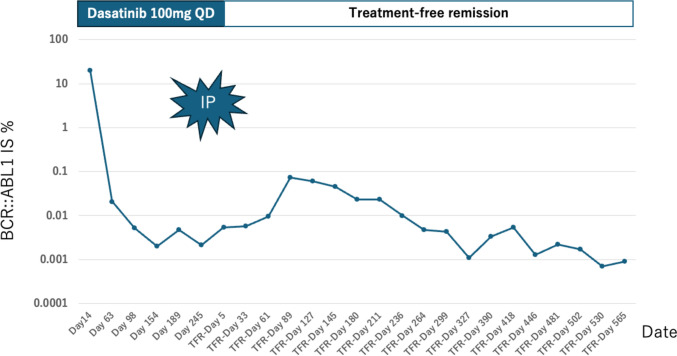
Clinical course and BCR::ABL1 (IS%) transcript kinetics. The entire clinical course, including dasatinib initiation, discontinuation due to adverse events, and subsequent long-term TFR, is summarized. BCR::ABL1 transcript levels declined rapidly after starting dasatinib, reaching MR^4.5^ (DMR) by day 154. Dasatinib was discontinued on day 282 due to the development of drug-induced interstitial pneumonitis (IP). Following discontinuation, the IS values showed a transient 1-log increase, peaking at 0.0729% on day 371 (TFR-Day 89). Following the peak, IS values spontaneously declined without resumption of TKI therapy (calculated halving time: 63 days). MR^4.5^ was reachieved 446 days after discontinuation, and DMR was maintained for over 7 years

Lymphocytosis was observed immediately after treatment initiation. The patient achieved a major molecular response (MMR, BCR::ABL1 International Scale [IS] ≤ 0.1%) at 3 months and MR^4.5^ (BCR::ABL1 IS ≤ 0.0032%) at 5 months. At 9 months (day 282), the patient developed a cough and dyspnea. Her SpO2 was 93% in room air. Computed tomography (CT) revealed diffuse interstitial opacities (Fig. 1). Cytomegalovirus (CMV) antibody titers were 86.6 AU/mL for immunoglobulin (Ig) G (normal range < 5.9) and 2.28 for IgM (normal range < 0.84), indicating a reactivation pattern; however, CMV antigenemia was not detected. Furthermore, the patient tested negative for antinuclear, anti-SSA/Ro, anti-Jo-1, and anti-neutrophil cytoplasmic antibodies. Although *β*-*D*-glucan was not examined, there was no evidence of infection or autoimmune disease. Based on these findings, dasatinib-induced interstitial pneumonia (Grade 3, Common Terminology Criteria for Adverse Events v5.0) was suspected. Dasatinib was immediately discontinued and prednisolone was administered, resulting in the resolution of pneumonia. When dasatinib was discontinued, the duration of the DMR was 128 days. Following discontinuation, BCR::ABL1 IS transcript levels transiently increased to 0.0729% on day 371 (TFR-Day 89). Although switching to another TKI was considered, the patient did not meet the criteria for resuming TKI therapy (loss of MMR). Considering the severe side effects and rapid molecular responses, we opted for careful monitoring and the transcript levels spontaneously declined without the resumption of TKI therapy. The patient reachieved MR^4^ on TFR-Day 236 and MR^4.5^ on TFR-Day 446, and she has remained in TFR with undetectable BCR::ABL1 mRNA for over 7 years (Fig. 2).

### Immunological analysis

**Fig. 3:**
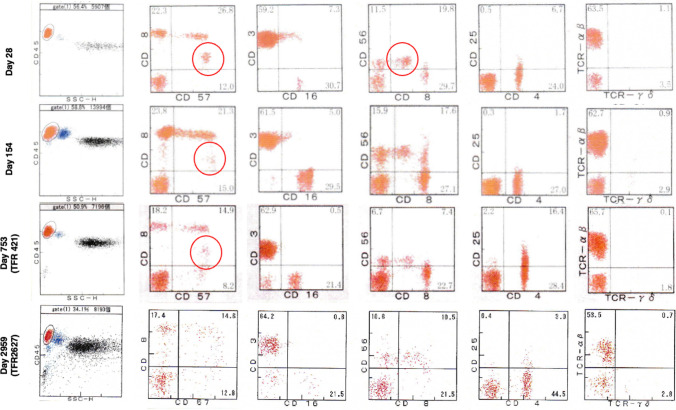
Longitudinal flow cytometric analysis of peripheral blood lymphocytes. The clinical course from the initiation of dasatinib treatment to the long-term treatment-free remission (TFR) phase is shown (days 28, 154, 753, and 2959). Day 28 (1 month after starting dasatinib): A significant increase in lymphocytes is observed. In the CD8 vs. CD57 cytogram, a unique cell population characterized by CD8^dim^ and CD57 positivity is observed (red circle). This population also expressed CD56, consistent with the phenotype of natural killer (NK)-like *T*-cells. Day 154 (attainment of DMR): The CD57^+^CD8^dim^ *T*-cell population remained detectable, although the clone size decreased compared to that on day 28. Day 753 (TFR-Day 421): More than 1 year after dasatinib discontinuation, this specific cell population (red circle) remained present, suggesting sustained endogenous immune surveillance. Day 2959 (TFR-Day 2,627): During the long-term TFR maintenance phase, the CD8^dim^ fraction and over-expansion of CD56/CD57-positive cells were resolved, and the lymphocyte profile returned to a pattern similar to that of healthy individuals

**Fig. 4:**
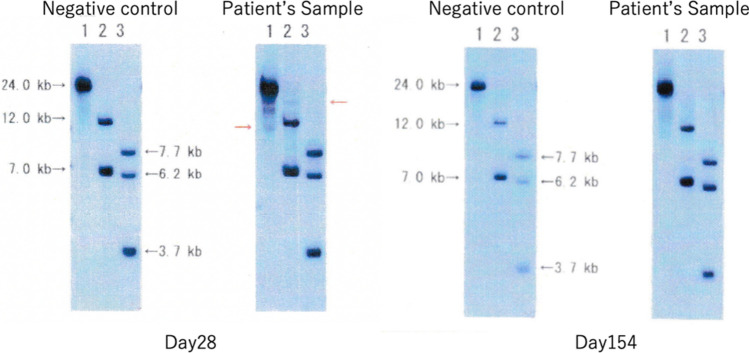
T-cell receptor (TCR) Cβ1 gene rearrangement analysis. The clonality of peripheral blood mononuclear cells was evaluated using Southern blotting. Day 28: A clear clonal band (arrow) indicating a TCR rearrangement was observed. The rearranged bands were identical in size after digestion with BamHI and EcoRV (lane 1: BamHI; lane 2: EcoRV; and lane 3: HindIII). Day 154: At the time of the deep molecular response (DMR, IS 0.0020%), the rearrangement band observed on day 28 disappeared, indicating that the clone size had decreased below the detection limit

Longitudinal immunophenotyping and T-cell receptor (TCR) gene rearrangement analyses were performed to elucidate the immune-mediated mechanisms contributing to the sustained TFR (Table 2). On day 28, during the early phase of dasatinib therapy, the patient exhibited significant lymphocytosis (3,779/μL), with T-cells (CD3^+^) accounting for 66.5% of white blood cells. Notably, the majority were CD8^+^ T-cells (49%), of which a distinct population of CD56^+^CD57^+^CD8^dim^ T-cells was identified (Fig. 3). Southern blot analysis on day 28 revealed a clear clonal band in the TCR Cβ1 gene, confirming the monoclonal expansion of this T-cell subset (Fig. 4). By day 154, although the CD56^+^CD57^+^CD8^dim^ population persisted, the clone size had diminished and the TCR rearrangement band was no longer detectable. After discontinuation of dasatinib, the absolute lymphocyte count and CD8^+^ effector fraction remained relatively stable. On TFR-Day 421, while the BCR::ABL1 levels showed minor fluctuations, the CD56^+^CD57^+^CD8^dim^ *T*-cell fraction was still observable via flow cytometry (FCM). At the most recent follow-up (TFR-Day 2,627), the lymphocyte subsets were normalized, with no evidence of clonal *T*-cell expansion or TCR rearrangement.

**Table 2:** Longitudinal changes in lymphocyte subsets and TCR rearrangement

	Day 28	Day 154	Day 282 (stop)	TFR-Day 421	TFR-Day 2627
WBC (/μL)	6,700	10,800	11,000	6,500	5,500
Hb (g/dL)	12.8	12.7	13.4	14.7	13.5
Plt (× 10^9^ /L)	141	111	151	251	222
Lym (%)	56.4	58.8	45	50.9	34.1
Lym (/μL)	3,779	6,350	4950	3,309	1,876
CD3 + (/μL)	2,513	3,582	NA	2,382	1,204
CD57 + CD8 + (/μL)	1,013	1,353	NA	493	274
CD56 + CD8 + (/μL)	748	1,118	NA	245	401
CD4 + CD25 + (/μL)	253	108	NA	543	56
TCR rearrangement	(+)	(-)	NA	(-)	(-)

WBC, white blood cells; Hb, hemoglobin; PLT, platelets; LYM, lymphocytes; ABS, absolute lymphocyte count; CD, cluster of differentiation; TCR, *T*-cell receptor; TFR, treatment-free remission; NA, not available

## Discussion

This case provides two clinical insights that challenge the current paradigm of TFR in CML. First, it demonstrated that long-term TFR can be achieved even after an exceptionally short duration of dasatinib therapy (9 months) and a minimal DMR duration (128 days). Second, this suggests that the clonal expansion of a unique *T*-cell subset, CD56^+^CD57^+^CD8^dim^ *T*-cells, may serve as a potent mediator of immune surveillance, potentially compensating for the lack of prolonged TKI-induced leukemia suppression.

According to current guidelines, such as those from the NCCN, ELN, and JSH, “eligibility” for TKI discontinuation typically requires at least 3 years of TKI therapy and sustained DMR (MR^4^ or deeper) for at least 2 years [3–5]. These criteria assume that prolonged TKI exposure is necessary to reduce the LSC pool to a level that can be controlled by the immune system. However, our patient discontinued dasatinib after 9 months because of grade 3 interstitial pneumonitis. Interestingly, despite a transient rebound in BCR: ABL1 transcripts (IS 0.0729%) shortly after cessation, the levels subsequently declined spontaneously. During the initial re-elevation phase, the calculated doubling time of the BCR: ABL1 transcript was 23 days. According to the criteria reported by Kim et al. [10], a doubling time of 23 days falls within the “intermediate-risk” group for molecular relapse (defined as 21–35 days), suggesting a significant risk of progressing to a full molecular recurrence. In most cases within this risk category, MMR loss is anticipated and the resumption of TKI therapy is typically required. Nevertheless, in our patient, the transcript levels spontaneously began to decline without any pharmacological intervention, with a subsequent “halving time” of 63 days. This is notably slower than the rapid decline typically seen with TKI therapy (average of 14 days) [11]; however, it represents a steady clearance of the residual clone in the absence of any pharmacological intervention. This “*V*-shaped” kinetics—rebound followed by an endogenous decline—strongly supports the presence of a robust, TKI-independent immune-mediated anti-leukemic effect.

Mustjoki et al. conducted a clinical study involving 22 patients who developed large granular lymphocytes (LGL) during dasatinib therapy [12]. These patients showed marked lymphocytosis with LGL morphology at a median of 3 months (range, 1–15 months) after the initiation of dasatinib treatment. Although 18 of the 22 patients experienced treatment-related complications, such as pleural effusion, colitis, or pneumonia following lymphocytosis, favorable clinical responses for CML were ultimately achieved in all cases. These LGLs were categorized into T-LGL (CD8^+^CD57^+^) and NK-LGL (CD16^+^CD56^+^), with TCRγδ rearrangement identified in all T-LGL cases. Furthermore, Kreutzman et al. suggested that the expansion of T-LGLs and NK-LGL during dasatinib therapy was associated with CMV reactivation [13]. They reported that persistent CMV reactivation during dasatinib treatment triggered clonal expansion of cytotoxic T/NK cells with a high cytokine-producing capacity. In some patients, T-cells that specifically recognize the leukemia-associated antigen PR1 have been detected. In the present case, although the LGL morphology was not formally confirmed on a blood smear, FCM analysis during dasatinib-induced lymphocytosis strongly suggested that the CD8^+^CD57^+^ cells were T-LGLs, which is consistent with the phenomena described by Mustjoki and Kreutzman.

Recent studies have highlighted the role of “innate CD8^+^ *T*-cells” in the efficacy of TKI therapy in CML [14]. Innate CD8^+^ *T*-cells, a subset recently proposed by Barbarin et al., are an NK-like CD8^+^ *T*-cell population that expresses NK cell surface markers such as KIR and NKG2A. These cells possess potent anti-tumor activity and rapidly produce IFN-*γ* in response to innate immune stimuli such as interleukin (IL)-12 and IL-18. In a study of 38 newly diagnosed CML cases, Barbarin et al. demonstrated that innate CD8^+^ *T*-cells increased significantly after 3 months of dasatinib therapy compared to baseline, followed by a gradual decrease in their proportion thereafter. Moreover, the proportion of these cells was significantly higher in patients who achieved DMR than in those who did not [15].

In our patient, an increase in CD8^+^CD57^+^ cells was observed during dasatinib therapy, comprising two distinct populations: CD56^+^CD57^+^CD8^dim^ *T*-cells and CD56^+^ CD57^+^CD8^high^ T-cells. Based on the longitudinal FCM and TCR rearrangement findings, the CD56^+^CD57^+^CD8^dim^ *T*-cell cluster was considered a clonal NK-like *T*-cell population. The surface phenotype and the kinetic changes in this population closely resemble the “innate CD8^+^ *T*-cells” defined by Barbarin et al. It is plausible that these dasatinib-induced NK-like *T*-cells exerted innate-like anti-tumor effects during the early phase of treatment and subsequently provided a sustained “booster” effect against the transient rebound of the CML clone after dasatinib discontinuation.

While the CD56^+^CD57^+^CD8^dim^ *T*-cell clone became undetectable by TFR-Day 2,627, the patient maintained TFR with undetectable BCR::ABL1 mRNA throughout the 7-year follow-up. This suggests that the initial robust immune response might have effectively reduced the leukemic stem cell pool below a critical threshold or that immune surveillance shifted to a more conventional, non-clonal memory *T*-cell distribution. Unlike other TKIs, the unique ability of dasatinib to induce LGL expansion likely played a decisive role in the ultra-early TFR success.

The limitations of this study include the lack of functional assays or cytokine profiles for CD56^+^CD57^+^CD8^dim^ *T*-cells, and the fact that the targets of clonal *T*-cells, such as the leukemia-associated antigen PR1, were not investigated. Despite these limitations, the unique clinical course of this patient strongly suggests that the immunological anti-leukemic effects mediated by NK-like *T*-cells significantly contributed to the successful long-term maintenance of TFR.

In conclusion, we reported a rare case of ultra-early TFR success mediated by the clonal expansion of *T*-cells, probably CD56^+^CD57^+^CD8^dim^ NK-like *T*-cells. The presence of these unique immune signatures may provide new avenues for identifying candidates for the safe discontinuation of TKIs.

## Declarations

### Data availability

No datasets were generated or analyzed during the current study.
